# A Quest to Find the Aetiology of Pulmonary Embolism Beyond the Common: A Case of Dyshypofibrinogenemia Presenting as Pulmonary Embolism

**DOI:** 10.7759/cureus.37647

**Published:** 2023-04-16

**Authors:** Saquib Siddiqui, Umair Falak

**Affiliations:** 1 Respiratory Medicine, Queen Elizabeth Hospital, Gateshead, GBR

**Keywords:** dyshypofibrinogenemia, congenital fibrinogen disorder, hypodysfibrinogenemia, pulmonary embolism (pe), acute pulmonary embolism

## Abstract

Hypodysfibrinogenemia-related thromboembolic disorder is a rarely encountered clinical entity. We present such a case of a 34-year-old lady with no known co-morbidities presenting to the accident and emergency unit with left-sided pleuritic chest pain associated with non-productive cough and breathlessness. Laboratory tests revealed fibrinogen level of 0.42 g/l (1.5-4g/l) with prolonged prothrombin time (PT), activated partial thromboplastin time (aPTT) along with elevated d-dimer, N-terminal pro-B-type natriuretic peptide (NT-proBNP), and troponin. CT pulmonary angiogram (CTPA) found bilateral pulmonary embolism with right heart strain. Functional/antigenic fibrinogen ratio was 0.38. Genetic testing eventually revealed a heterozygous missense mutation in exon 8-p.1055G>C; p.Cys352Ser in the sequencing of the fibrinogen gene FGG (gamma chain) confirming the diagnosis of dyshypofibrinogenemia. She was treated with anticoagulants with fibrinogen replacement therapy and later discharged on apixaban.

## Introduction

Congenital fibrinogen disorder such as dyshypofibrinogenemia is a rarely encountered clinical entity that can present with both bleeding and thrombotic events. Managing such patients with thrombosis is always challenging as anticoagulant treatment has the potential to result in bleeding leading to catastrophic outcome. There is very limited information from trials about management of this condition due to low prevalence. We present one such case of sub-massive pulmonary embolism resulting from dyshypofibrinogenemia. 

## Case presentation

A 34-year-old woman with no known co-morbidities presented to the Emergency Department with left-sided pleuritic chest pain. On further query she also mentioned non-productive cough and shortness of breath for a week that got worse over the past 24 hours. 

During general examination, she was noted to be markedly dyspnoeic, tachycardic, and tachypnoeic. Her oxygen saturation was 92-95% on 4L nasal cannula. Chest auscultation revealed clear lung fields. A 12-lead electrocardiogram showed sinus tachycardia without any evidence of arrhythmia. Arterial blood gas showed hypoxic respiratory failure with a ph of 7.58, partial pressure of oxygen (PO2) 8.9, partial pressure of carbon dioxide (PCO2) 4.3, and bicarbonate (HCO3) 24 (on 4L nasal cannula).

**Figure 1:**
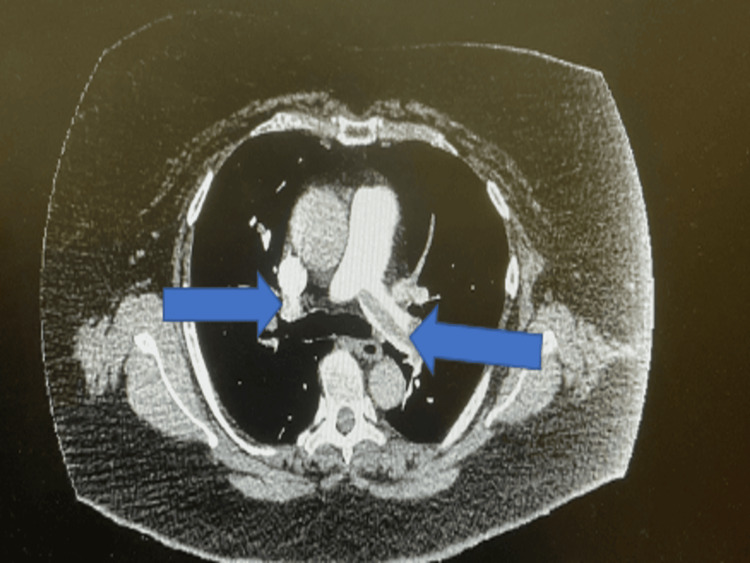
Significant bilateral pulmonary embolism (blue arrows)

**Figure 2:**
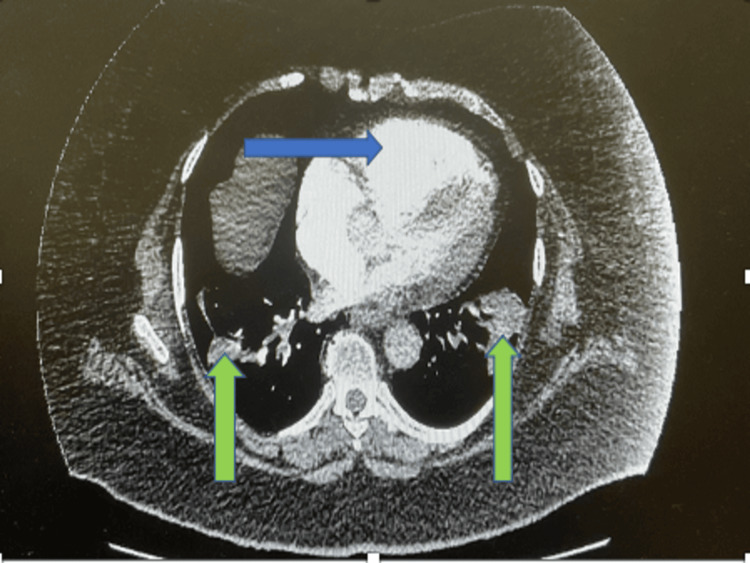
Right heart strain (blue arrow) with pulmonary embolism with pulmonary infarction (green arrows)

The laboratory results revealed elevated levels of inflammatory markers with high C-reactive protein and neutrophilic leucocytosis. Coagulation profile showed low fibrinogen level along with prolonged thrombin time and a raised d-dimer (Table 1). A severe acute respiratory syndrome coronavirus 2 (SARS-CoV-2) test for covid was negative. CT pulmonary angiogram was performed as Wells’ score for pulmonary embolism was 4.5 along with a positive d-dimer. CT pulmonary angiogram revealed significant bi-lateral pulmonary embolism associated with right heart strain (Figures 1, 2). Therefore NT-proBNP, troponin, and echocardiogram were performed for risk stratification. Her NT-proBNP and troponin both were elevated (Table 1).

**Table 1:** Laboratory Outcome

Marker	Result	Reference Value
C-reactive protein	87 mg/l	0.3-1mg/l
White blood cells	12.4 × 103/μl	4-11 × 103/μl
Fibrinogen level	0.42 g/l	1.5-4.0 g/l
Thrombin time	18.9 seconds	11-15 seconds
D-dimer	20 μg/mlFEU	<0.5 μg/mlFEU
NT-proBNP	673 ng/l	0-100 ng/l
Troponin	47ng/l	0-13 ng/l

Transthoracic echocardiogram showed dilated right heart with reduced systolic function and raised right ventricular systolic pressure (RVSP) (47 mm hg) along with trivial mitral regurgitation and mild tricuspid regurgitation. She was classified as a case of sub-massive pulmonary embolism or intermediate high-risk pulmonary embolism (normotensive with elevated NT-proBNP, troponin with a pulmonary embolism severity index score of 4 along with CTPA and echocardiographic evidence of right ventricular enlargement/dysfunction).

She was started on a therapeutic dose of low molecular weight heparin (LMWH). Her blood pressure remained stable. Therefore, thrombolytic therapy was not required. She had a positive family history of blood clots as her mother also suffered from right-sided deep vein thrombosis. Due to presence of pulmonary embolism with significantly low fibrinogen levels, it was thought to rule out inherited fibrinogen disorder. Functional fibrinogen level (Clauss method) was 0.50g/L, antigenic fibrinogen level was 0.13g/L. Fibrinogen level with a functional/antigenic ratio was 0.38 (cut off 0.7). Finally the diagnosis was clinched with genetic testing which revealed a heterogenous missense mutation in exon 8-p.1055G>C; p.Cys352Ser in the sequencing of the fibrinogen gene FGG (gamma chain). 

After a discussion with Haematology about the genetic testing results, she continued to be treated with a therapeutic dose of enoxaparin along with replacement therapy in the form of stat dose of cryoprecipitate and fibrinogen concentrate. Thrombophilia and antiphospholipid syndrome screening were negative. She was later discharged on apixaban as declined to have warfarin with follow-up as an outpatient with Haematology.

## Discussion

Inherited/congenital fibrinogen disorders, a rare heterogeneous group of disorders first introduced in 1965, are associated with inherited abnormalities of coagulation with their frequency being as low as 0.80-0.91% [1]. Until then more than 300 mutations have been identified in FGA (fibrinogen alpha), FGB (fibrinogen beta), and FGG (fibrinogen gamma) genes. It is an autosomal dominant condition caused by heterozygosity for missense mutations of various fibrinogen genes such as FGA, FGB, and FGG [2]. Congenital fibrinogen disorders are classified into type 1 disorders primarily called afibrinogenemia, and hypofibrinogenemia which primarily affects the amount of fibrinogen in blood and type 2 disorders primarily called dysfibrinogenemia and dyshypofibrinogenemia which impact the quality of circulating fibrinogen. Dyshypofibrinogenemia, therefore shares features of both hypo- and dysfibrinogenemia. It can be subdivided according to fibrinogen levels too such as 1) severe hypodysfibrinogenemia (below 0.5 g/l); 2) moderate hypodysfibrinogenemia (0.5 - 0.9 g/l); 3) mild hypodysfibrinogenemia (1.0 g/l -1.5g/l).

Laboratory diagnostic criteria usually include low levels of circulating fibrinogen with prolonged/normal aPTT, PT, and thrombin time (TT) [3,4]. Another important laboratory finding is reduced ratio of functionally detected fibrinogen to immunoassay-detected fibrinogen (<0.7). It has a diagnostic sensitivity of 86% for dyshypofibrinogenemia. Eventually, detection of a mutation in the fibrinogen gene during genetic analysis remains the gold standard.

It can present as asymptomatic (55%), bleeding (25%), and thromboembolic events (20%). Venous thrombosis appears to be the most common thrombotic event particularly in hypofibrinogenemic patients [5-7]. The true mechanism(s) of thrombosis in inherited fibrinogen disorder remains unknown. A possible suggested mechanism includes elevated levels of thrombin and stability of the fibrin clot, as well as impaired fibrinolysis [8-10]. Mutations in FGA p.Arg573Cys, FGG p.Arg301His, and FGB P.arg478Lys are known to be of high thrombotic risk [2,11,12].

The principle of managing thromboembolic events in patients with fibrinogen disorders is the same as that in individuals presenting with clots for any other reasons. As per UK Haemophillia Centre Doctor’s organisation, anticoagulation with LMWH is the mainstay of treatment [13]. Warfarin remains an option as longer-term anti-coagulation. There is no existing study with direct oral anticoagulants (DOAC) but they are not known to cause any complications in such patients. Often fibrinogen replacement products may be required. Although no evidence exists on its use resulting in increased risk of thrombosis in such patients, we still would suggest a degree of caution is warranted while using fibrinogen replacement therapy. 

## Conclusions

Importance of giving emphasis on the coagulation profile of patients presenting with pulmonary thromboembolic disorders with having intuition to investigate for inherited fibrinogen disorders with low fibrinogen levels. Anticoagulation therapy with low molecular weight heparin is the mainstay of treatment of thrombotic events in such patients. It is always important to discuss with Haematology for further genetic testing along with requirement of fibrinogen replacement therapy in such patients. According to some reports, oral anticoagulants such as warfarin or DOAC can be used as long-term anticoagulants.
